# Lymph Node Stromal Cells From Different Draining Areas Distinctly Regulate the Development of Chronic Intestinal Inflammation

**DOI:** 10.3389/fimmu.2020.549473

**Published:** 2021-02-16

**Authors:** Marijana Basic, Pia Pascale Peppermüller, Silvia Bolsega, André Bleich, Melanie Bornemann, Ulrike Bode, Manuela Buettner

**Affiliations:** ^1^ Institute for Laboratory Animal Science, Hannover Medical School, Hannover, Germany; ^2^ Institute for Functional and Applied Anatomy, Hannover Medical School, Hannover, Germany

**Keywords:** cytokines, stromal cells, lymph nodes, transplantation, chemokine

## Abstract

The balance between the responsiveness of the intestinal immune system and the gut environment is fundamental for the maintenance of intestinal homeostasis, which is required for an adequate recognition of entering antigens. The disruption of this homeostasis by exaggerated immune response to harmless antigens can lead to the development of intestinal disorders such as inflammatory bowel disease. Stromal cells are sessile non-hematopoietic cells that build the backbone of the lymph node, an important site for the immune response induction, but also contribute to immune response and tolerance induction. However, the knowledge about the role of stromal cells in the regulation of inflammatory responses is still limited. Therefore, in this study we analyzed the influence of stromal cells on the development of chronic intestinal inflammation. Here, we show that intestinal inflammation alters the immune activation of the mesenteric lymph node-derived stromal cells. Podoplanin^+^ and CD21/35^+^ stromal cells showed increased expression of MHC class II molecules, but CD106 expression on CD21/35^+^ cells was reduced. Stromal cells secreted cytokines and chemokines such as CCL7 and CXCL16 influenced the gut-homing phenotype and proliferation of CD4^+^ and CD8^+^ T cells. Furthermore, stromal cells of peripheral lymph nodes transplanted into the mesentery attenuated colitis severity in B6-*Il10^-/-^* mice. The reduced colitis severity in these mice was associated with increased expression of IL4 and distinct activation pattern of stromal cells derived from transplanted peripheral lymph nodes. Altogether, our results demonstrate that lymph node stromal cells impact development of chronic colitis via T cell induction. Moreover, lymph node stromal cells from different draining area due to neonatally imprinted processes distinctly regulate the induction of immune responses.

## Introduction

The mucosal surface of the gut is the first contact and recognition site for many antigens (Ag). The intestinal homeostasis is required for an efficient decision whether entering Ag is harmless, such as food, or potentially pathogenic. To ensure the maintenance of the intestinal homeostasis, the host protective immunity and the gut microbiota have to be in balance. Disruption of this balance by exaggerated immune reaction to harmless antigens can lead to intestinal disorders such as inflammatory bowel disease (IBD). IBD is a chronic inflammatory disease of the gastrointestinal tract that encompasses Crohn’s disease and ulcerative colitis. Interleukin 10-deficient mouse model (*Il10^-/-^*) is a genetically engineered model for IBD research (1). Histopathological hallmarks of colitis in *Il10^-/-^* mice are inflammatory cell infiltration of the lamina propria and submucosa, epithelial hyperplasia, mucin depletion, crypt abscesses, ulceration, and thickening of the intestinal wall (2). Colitis development in mice carrying mutation in *Il10* gene starts shortly after weaning and is microbiota dependent, as germ-free mice do not develop any signs of inflammation (3, 4).

Lymph nodes (LN) are located at sites where pathogenic Ag might invade the host. They filter the lymph coming from the draining area and initiate immune responses. Every LN consists of mobile immune cells, e.g., dendritic cells (DCs) and lymphocytes, as well as sessile non-hematopoietic stromal cells (SCs) such as fibroblastic reticular cells (FRC) and follicular dendritic cells (FDC) (5, 6). Differences between the microenvironments of distinct LN are necessary to ensure an efficient immune response for the specific draining area of the LN. DCs and stromal cells of the mesenteric lymph nodes (mLN) preferentially express the retinal dehydrogenase 2 (RALDH2) (7–9). RALDH2 enzyme is involved in imprinting gut-homing specificity on T and B cells by upregulating the expression of gut-homing receptor CCR9 (10, 11). Plasma cells in the mLN were found to predominantly produce IgA, whereas plasma cells induced in peripheral lymph nodes (pLN) were mainly IgG producing cells (12). Microarray-, RNAseq-, and protein analyses revealed various expression differences between stromal cells draining different areas, but also between distinct stromal cell subpopulations (13–17). All these variations result normally in an optimal immune response to protect the specific draining area of the LN. A lymph node transplantation model, in which peripheral lymph node fragments are transplanted into the mesentery, is a valuable tool to identify the influence of stromal cells on immune response and tolerance induction (12, 18, 19). The transplanted lymph nodes (LNtx) regenerate within eight weeks to a fully functional LN repopulated with recipient-derived immune cells and donor-derived stromal cells (7, 18). Using this model, we were able to analyze the influence of stromal cells on chronic inflammatory diseases. In this study, mLN-derived cells from inflamed B6-*Il10^−/-^* mice were analyzed for their cytokine and chemokine expression pattern. Stimulation of T cells with cytokines and chemokines produced by stromal cells influenced their gut-homing phenotype and proliferation. Furthermore, stromal cells of transplanted peripheral lymph nodes (pLNtx) reduced colitis severity in B6-*Il10^-/-^* mice. In conclusion, lymph node stromal cells are able to influence the development of chronic inflammation.

## Materials and Methods

### Animals

C57BL/6J.129P2-*Il10* ^t^ *^m1Cgn^* (B6-*Il10* ^-/-^) were bred at the Central Animal Facility of the Hannover Medical School and were used at a weight of 18–25 g. Mice were housed in filter-top cages located in a room with a controlled environment and 12 hour light/dark cycle. If not stated otherwise, mice received pelleted diet (Altromin 1324 TPF, Altromin Spezialfutter GmbH & Co. KG, Lage, Germany) and autoclaved water *ad libitum*. Animals were monitored according to FELASA recommendations (20) and were proven to be free of infection with common murine pathogens except *Pasteurella pneumotropica*, *Helicobacter hepaticus* respectively *bilis*, β-haemolytic streptococci, *Klebsiella oxytoca*, *Staphylococcus aureus*, murine norovirus, *Trichomonas* spp., and apathogenic intestinal flagellates. Mice were sacrificed by cervical dislocation at 12 or 20 weeks of age.

### Ethical Statement

This study was conducted in accordance with German law for animal protection and with the European Directive 2010/63/EU. All experiments were approved by the Local Institutional Animal Care and Research Advisory committee and permitted by the local government (No. 09/1667, 11/0643, and 20/3451).

### Intestinal Surgery and LN Transplantation

mLN and pLN from B6-*Il10* ^-/-^ donor mice were isolated and disrupted. Under the combined anesthesia with ketamine (Gräub AG, Bern, Switzerland) and Domitor (Pfizer, Karlsruhe, Germany) all mLN of the small and large intestine from recipient B6-*Il10^-/-^* mice were excised and previously isolated mLN or pLN (axillary, brachial, popliteal, and inguinal LN) from donor mice were transplanted into the mesentery (12). The recipients were sacrificed 8 weeks after transplantation and the transplanted LN (LNtx) were analyzed.

### Chronic Colitis Induction

Chronic colitis was induced using 3.3% DSS (MP Biomedicals, Eschwege, Germany) in the drinking water for 4 days. Four weeks after DSS-induced colitis, mice were sacrificed and the LN were analyzed.

### Preparation of a Single-Cell Suspension From the Small Intestine

The small intestine was removed and rinsed with PBS. Subsequently, Peyer’s patches (PPs) were removed and the small intestine was cut open and sliced into thick section slices. These pieces were minced and then placed into a Hanks Salt solution (Biochrom) containing 2 mM EDTA (Serva, Heidelberg, Germany), 1 mM DL-Dithiothreitol (Sigma), and 5% FCS for 20 minutes at 37°C. The suspension was subsequently filtered. This procedure was repeated three times. These first cell suspensions including epithelial cells were collected and pooled. Remaining slices were incubated in Hanks Salt solution containing 1.5 mg/ml collagenease VIII (Sigma-Aldrich, St. Louis, USA) and 5% FCS. This second cell suspension was also filtered and added to the first collected cell suspension. Cells were collected by centrifugation. After centrifugation cells were counted and analyzed by flow cytometry.

### Preparation of a Single-Cell Suspension From mLN

The whole mLN string of B6-*Il10* ^-/-^ mice were removed and LN were digested at 37°C for 30 min with 1 mg/ml collagenase VIII (Sigma-Aldrich) in RPMI 1640 containing 10% FCS. CD45^-^ cells were isolated using the MACS technique following the instructions provided by Miltenyi (Bergisch-Gladbach, Germany).

### Isolation of mLN Stromal Cells

For isolation of CD45- SCs, the small intestine draining mLN (simLN) or the colon draining mLN (cmLN) of macroscopically healthy and colitongenic B6-*Il10^-/-^* mice were removed as described recently (21, 22). LN were digested at 37°C for 30 min with 1 mg/ml collagenase VIII (Sigma-Aldrich) in RPMI 1640/10% FCS. CD45- cells were isolated using the MACS technique following the instructions provided by Miltenyi (Bergisch-Gladbach, Germany). The isolated SCs were used for mRNA isolation.

### Isolation and Stimulation of T Cells

Cell suspensions from the spleen of B6-*Il10* ^-/-^ mice were made and erythrocytes were lysed as described previously (23). Splenic T cells were isolated by negative selection using magnetic cell separation. Therefore, cell suspension was incubated with anti-MHCII (BD Biosciences) and IgG beads (MACS, Miltenyi) each for 20 min. Isolated cells were stained with CFSE (5 mmol) for 2 min and washed with MACS buffer. Next, 2x10^6^ cells/well were cultured with or without anti-CD3 antibody (Biolegend) for 48 hours in RPMI medium (Biochrom, Berlin, Germany) with 10% FCS (GE Healthcare Life Sciences, Buckinghamshire, UK), 1% Pen/Strep (Gibco, Thermo Fisher Scientific, Waltham, USA), 0.3 mg/ml Glutamin (Biochrom), and 10 µM β-mercaptoethanol containing recombinant protein (770 ng/mL CCL5/RANTES 134 ng/mL CCL2, 200 ng/mL CCL7, and 150 ng/mL CXCL16; all Peprotech) for stimulation. Stimulation experiments were performed in duplex and data were generated from 2–5 independent experiments.

### Histology

Colon samples were fixed in neutral buffered 4% formalin, embedded in paraffin, sectioned at 5–6 µm, and stained with hematoxylin and eosin. Histology slides were blindly scored for ulceration, hyperplasia, severity, and the involved area as described previously (2). Briefly, each parameter was graded from physiological (0) to severe changes (3) and added in a total score from 0 to 12. Colon sections were scored separately for the proximal, middle, and distal part. A total colon score was calculated by adding all three colon sections.

### Antibodies for Flow Cytometry

Stromal cells were analyzed using anti-CD45-PE-Cy7, anti-CD21/35-APC (BD Biosciences, Heidelberg, Germany), anti-Podoplanin-FITC (gp38; Biozol, Eching, Germany), anti-CD31-APC (Biolegend, San Diego, USA), and anti-Lyve-1 (kindly provided by R. Förster, Institute of Immunology, Hannover Medical School, Germany) detected by a Cy3-coupled goat anti-rabbit antibody (Dianova, Hamburg, Germany). Activation of stromal cells was determined using anti-MHCII-PE, anti-CD54-PE (BD Biosciences), and anti-CD106-PE (Serotec).

Cell suspensions from mLN and the small intestine were prepared as described above. In addition, 1x10^6^ cells from mLN and the small intestine were incubated with anti-CD3-FITC, anti-F4/80-APC (both from Biolegend), anti-CD8-PE-Cy7, anti-CD4-APC, anti-IgA-PE (all from Serotec, Oxford, UK), anti-CD19-APC-H7, anti-LPAM1-PE, anti-CD11c-FITC (all acquired from BD Biosciences), and anti-CCR9-PE (eBiosciences). All FACS analyses were performed on a FACSCanto (BD Biosciences) and analyzed using Diva software (BD Biosciences) or Kaluza software (Beckmann Coulter).

### Quantification of mRNA Expression

The total RNA was isolated from snap frozen mLN according to the manufacturer’s protocol (Bioline, London, UK). The cDNA synthesis was performed with 50 µM oligo primer, 0.1 M DTT, 5x first strand buffer, 10 mM dNTP mix, 40 U/µl Rnase inhibitor, and 200 U/µl Superscript III reverse transcriptase (all obtained from Invitrogen) in a total volume of 20 µl at 50°C for 50 min. Obtained cDNA was used for quantitative real time PCR (qPCR). For SYBR Green^®^ chemistry analyses QuantiTect SYBR Green protocol from Qiagen was used according to the manufacturer’s recommendations. The primer sequences and amplicon sizes of *Tnf*a (5´- GACCCTCACACTCAGATCATCTTC -3´ and 5´- CGCTGGCTCAGCCACTCC -3´; 104 bp), *Il4* (5´- ACGAGGTCACAGGAGAAGGGA -3´ and 5´- AGCCCTACAGACGAGCTCACTC -3´; 129 bp), and β-actin (5´- AGCCATGTACGTAGCCATCC -3´ and 5´- CTCTCAGCTGTGGTGGTGAA -3´; 228 bp) were used. QPCR analyses were performed in CFX Real Time PCR Detection System (Biorad). The amplified PCR product was verified by melting curve analysis. TaqMan^®^ chemistry analyses were performed using TaqMan^®^ Gene Expression Assays (ThermoFisher Scientific) for *Ccl7* (Mm00443113_m1), *Ccl2* (Mm00441242_m1), *Cxcl16* (Mm00469712_m1), *Ccl5* (Mm01302427_m1), and β-actin (Mm_00607939_s1). Detection was performed with QuantStudio 6 Flex Real-Time PCR System (Applied Biosystems, Weiterstadt, Germany) using the TaqMan® Fast Advanced Master Mix according to the manufacturer’s instruction. β-actin was used as a reference gene in both assays. All reactions were run in triplicate. Relative gene expression was calculated using the 2^−ΔCt^ method if not stated otherwise.

### Magnetic Cytokine Assays

The cytokine levels of IL1b, IL2, IL4, IL5, IL6, IL12(p40), IL12(p70), IL13, IL17, G-CSF, IFNg, MCP1, RANTES, and TNFa were measured in the supernatants of T cell stimulation experiments or in serum using Bio-Plex Pro cytokine assays (Bio-Rad, Hercules, USA). The assay was performed according to the manufacturer’s instructions.

### Data Analysis

Calculations, statistical analysis, and graphs were made with the software Graphpad Prism 8.0 (Graphpad Software Inc., San Diego, USA). Statistical differences were calculated using unpaired t-test when comparing two groups and one-way analysis of variance (ANOVA) with Tukey’s test for multiple comparisons when comparing three or more groups. Statistically significant differences are indicated by *, P< 0.05; **, P< 0.01; ***, P< 0.001; ****, P< 0.0001.

## Results

### Intestinal Inflammation Alters Immune Activation of mLN Stromal Cells

**Figure 1:**
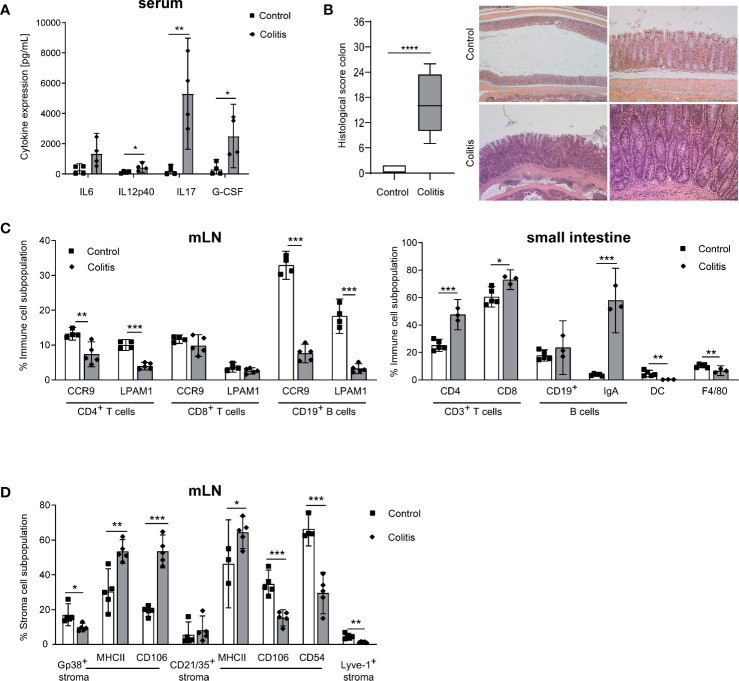
Impact of chronic intestinal inflammation on the gut and mLN cell compartments. **(A)** Levels of pro-inflammatory cytokines measured in serum of control and inflamed B6-*Il10^-/-^* mice (filled squares: control, filled diamonds: colitis). Data were shown as mean ± 95% confidential intervals (n=4). **(B)** Histopathological score quantifying alterations observed in the colon of B6-*Il10^-/-^* control and inflamed (animals in which chronic colitis was triggered by DSS) mice. Data presented in box and whiskers plots are the medians with minimum and maximum (n=5–7). Representative images of hematoxylin and eosin stained colon sections of 12-week-old control (upper panels) and inflamed (lower panels) B6-*Il10^-/-^* mice (magnification left panels: 2.5x; magnification right panels: 10x). **(C)** Flow cytometry analysis of single cell suspensions from mLN and the small intestine of control and inflamed B6-*Il10^-/-^* mice (filled squares: control, filled diamonds: colitis). Plots summarize frequencies of immune cell populations in mLN (CD4^+^ T cells, CD8^+^ T cells, and CD19^+^ B cells) and the small intestine (CD3^+^ T cells, B cells, DCs and F4/80^+^ macrophages). Data were shown as mean ± 95% confidential intervals (n=3–5). **(D)** Flow cytometry analysis of single cell suspensions from mLN of control and inflamed B6-*Il10^-/-^* mice (filled squares: control, filled diamonds: colitis). Plots summarize frequencies of stromal cell populations (Gp38^+^, CD21/35^+^, and Lyve-1^+^ stromal cells). Data were shown as mean ± 95% confidential intervals (n=3–5). Statistically significant differences are indicated by *, P< 0.05; **, P< 0.01; ***, P< 0.001; ****, P< 0.0001.

The development of spontaneous colitis in *Il10* ^-/-^ mice strongly depends on the microbiota composition (3, 4). To cause a reproducible colitis onset in our model, mice were exposed to 3.3% DSS in the drinking water for four days. Four weeks after DSS-induced colitis onset, mice were sacrificed and colitis severity was analyzed. Various inflammatory parameters such as IL17, G-CSF, and IL12p40 were increased in serum pointing to a high-grade inflammation (**Figure 1A**). As the intestinal inflammation in *Il10^-/-^* mice mainly affects the large intestine, we analyzed histopathological changes in the colon. The colon of B6-*Il10^-/-^* mice was severely inflamed (**Figure 1B**). The intestinal pathology was characterized by severe infiltrations of immune cells up to L. muscularis, severe mucosal architecture loss, extended ulcerations, and peritonitis (**Figure 1B**). Furthermore, all gut draining LN and the small intestine were analyzed via flow cytometry (**Supplementary Figures 1** and **2**). The mLN showed decreased percentages of gut homing molecules on CD4^+^ T cells and B cells (**Figure 1C**). In the small intestine T cells as well as IgA^+^ B cells were increased, whereas DC and macrophages were decreased (**Figure 1C**). Furthermore, we detected decreased podoplanin^+^ (Gp38^+^) and also Lyve-1^+^ cells in mLN (**Figure 1D**). Podoplanin^+^ as well as CD21/35^+^ cells showed increased MHC class II expression suggesting that these cells are activated. Also CD106 was highly expressed on podoplanin^+^ cells. In contrast, CD21/35^+^ cells showed reduced expression of CD106 as well as CD54 (**Figure 1D**). Thus, these results showed that intestinal inflammation in B6-*Il10* ^-/-^ mice affects the whole intestinal tract including mLN and especially mLN stromal cells.

### CCL7 and CXCL16 Activate T Cells During Inflammation

**Figure 2:**
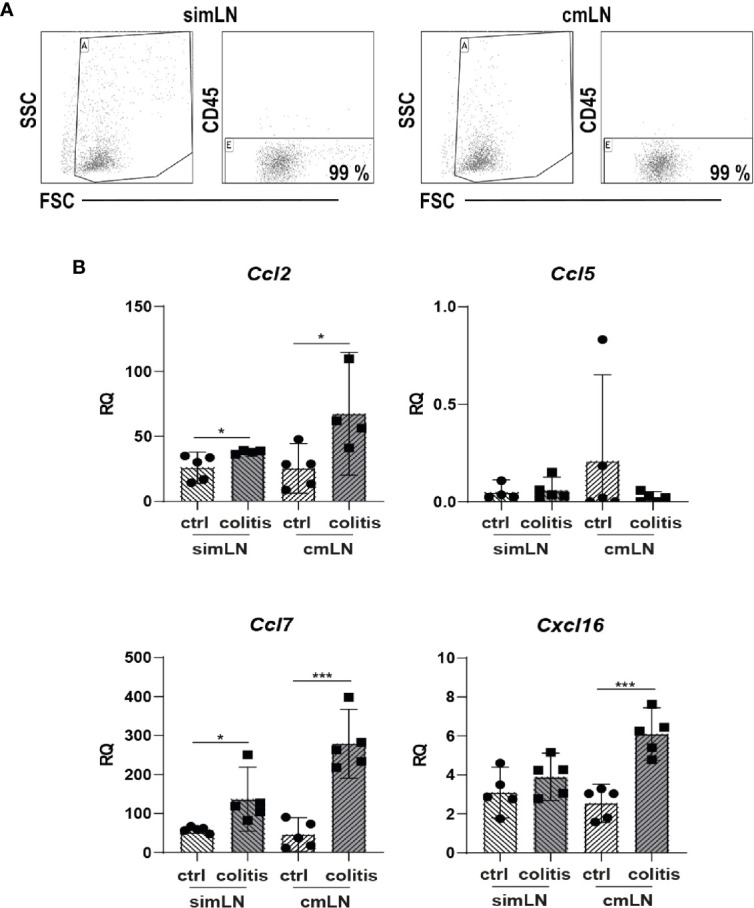
The intestinal inflammation alters expression of chemokines in mLN-derived stromal cells. Stromal cells were isolated from healthy and colitogenic small intestine draining or colon draining mLN. **(A)** Representative Dot Plots of CD45 negative cell enrichment obtained after selection procedure. **(B)** Gene expression of chemokines *Ccl2*, *Ccl5*, *Ccl7*, and *Cxcl16* measured by qPCR in total RNA isolated from small intestine draining or colon draining mLN stromal cells of control and inflamed B6-*Il10^-/-^* mice (filled circles: control, filled squares: colitis). Relative differences in gene expression were calculated by the relative quantification (RQ) method (2^−ΔΔCt^ method) using a reference sample and housekeeping gene for normalization of gene expression. Data were shown as mean ± 95% confidential intervals (n=4–5). Statistically significant differences are indicated by *, P< 0.05; ***, P< 0.001.

**Figure 3:**
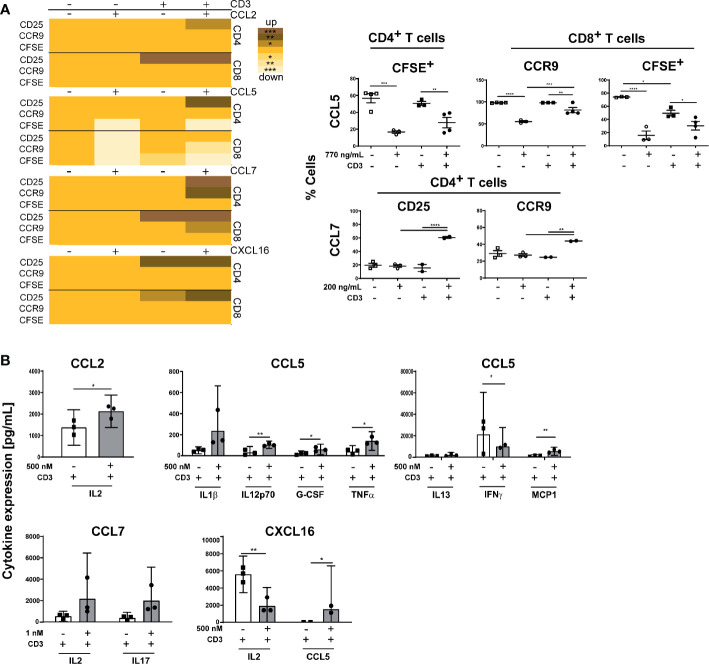
Chemokine stimulation influence activation and proliferation of B6-*Il10^-/-^* splenic T cells. **(A)** Isolated splenic T cells were stimulated with chemokines and/or CD3 for 48 hours and proliferation and expression of gut-homing molecules on CD4^+^ and CD8^+^ T cells was detected using flow cytometry. Heat map shows the significant differences of T cell activation and proliferation after CD3 and chemokine stimulation (CCL2, CCL5, CCL7, or CXCL16). Scatterplots, showing differences dependent on chemokine stimulation, summarize frequencies of CD25^+^, CCR9^+^, and CFSE^+^ T cells upon stimulation with chemokine, and co-stimulation with CD3 (open squares: control, open circles: chemokine stimulation, filled squares: CD3 stimulation, filled circles: CD3/chemokine co-stimulation). Plotted are mean values of two replicates from 2–5 independent experiments. Data were shown as mean ± SEM. **(B)** Levels of expressed cytokines/chemokines detected in the supernatant of T cells stimulated only with specific chemokine (CCL2, CCL5, CCL7, or CXCL16) or co-stimulated with CD3 isolated from B6-*Il10^-/-^* mice (filled squares: CD3 stimulation, filled circles: CD3/chemokine co-stimulation). Plotted are single values from 2–3 independent experiments. Data were shown as mean ± confidential intervals (n=2–3). Statistically significant differences are indicated by *, P< 0.05; **, P< 0.01; ***, P< 0.001; ****, P< 0.0001.

Various chemokines such as CCL2, CCL5, CCL7, or CXCL16, which are important for recruitment and organization of T cells and DC or after induction of inflammation, were shown to be produced by stromal cells (15, 16). Therefore, we measured the gene expression of these genes in CD45- stromal cells isolated from small intestine draining (si) mLN or colon draining (c) mLN of control and inflamed B6-*Il10^-/-^* mice by qPCR. The gene expression of *Ccl2*, *Ccl7*, and *Cxcl16* was increased in colitogenic cmLN stromal cells. Stromal cells isolated from simLN showed also differences in gene expression of *Ccl2* and *Ccl7*, although to a lower extent. Expression of *Cxcl16* was unchanged in simLN stromal cells of mice suffering from colonic inflammation (**Figure 2**). Expression of *Ccl5* was unaltered in healthy and colitogenic stromal cells isolated from simLN and cmLN. Subsequently, we isolated T cells from the spleen and cultured them without or with these chemokines (CCL2, CCL5, CCL7, and CXCL16) for 48 hours. Upon chemokine stimulation, activation (induction of gut homing molecules) and proliferation (CFSE staining) of T cells were detected using flow cytometry (**Supplementary Figure 3**). Stimulation experiments were performed in duplex and data were generated from 2–5 independent experiments. Each chemokine stimulation was performed independent from the others to exclude chemokine contamination. Upon CCL2 stimulation, no differences in T cell proliferation and activation dependent on CCL2 were found (**Figure 3A**). However, in the supernatant of CCL2 and CD3 co-stimulated T cells increased levels of IL2 were measured (**Figure 3B**). CD4^+^ and CD8^+^ T cells stimulated only with CCL5 showed decreased activation and proliferation. After co-stimulation with CD3, only proliferation in CD4^+^ T cells was decreased (**Figure 3A**). However, increased levels of TNFα, IL12p70, G-CSF, and MCP-1 (CCL2) in the supernatants of CCL5 and CD3 co-stimulated T cells were detected (**Figure 3B**). In addition, these cells showed decreased levels of IFN*γ* (**Figure 3B**). The co-stimulation of T cells with recombinant CCL7 and CD3 resulted in activated T cells. We detected higher percentages of CD25^+^ and CCR9^+^ CD4^+^ T cells (**Figure 3A**). These cells also produced higher levels of IL2 and IL17 as detected in the supernatants of the cultured cells (**Figure 3B**). Furthermore, we measured higher percentages of CFSE^+^ cells after stimulation with CXCL16/CD3 among CD8^+^ T cells (**Figure 3A**). In the supernatants of these co-stimulated cells, we measured increased levels of RANTES (CCL5) and decreased concentrations of IL2 (**Figure 3B**). Together, these results indicate that T cell activation could be influenced by chemokines produced by lymph node cells including stromal cells.

### PLN Stromal Cells Reduce Severity of Chronic Colitis

**Figure 4:**
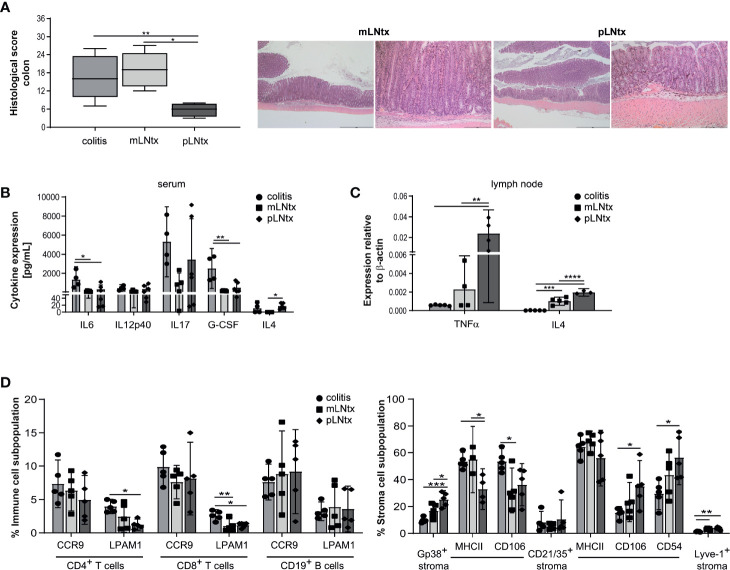
Stromal cells from pLN attenuate the severity of chronic colitis. **(A)** Histopathological score quantifying alterations observed in the colon tissue of 20-week-old B6-*Il10^-/-^* mice after transplantation of pLN (pLNtx) or mLN (mLNtx) into the mesentery and inflamed B6-*Il10^-/-^* mice (as already shown in **Figure 1A**). Data presented in box and whiskers plots are the medians with minimum and maximum (n=4–5). Representative images of hematoxylin and eosin stained colon sections of mLNtx and pLNtx B6-*Il10^−/-^* mice (mLNtx magnification left panel: 2.5x, right panel: 10x; pLNtx magnification left panel: 2.5x, right panel: 10x). **(B)** Levels of pro-inflammatory cytokines measured in serum of inflamed B6- *Il10^-/-^* mice and mLNtx and pLNtx B6-*Il10^-/-^* mice (filled circles: colitis, filled squares: mLNtx, filled diamonds: pLNtx). Data were shown as mean ± 95% confidential intervals (n=3–5). **(C)** Gene expression of *Tnfa* and *Il4* measured by qPCR in total RNA isolated from mLN of inflamed B6-*Il10^-/-^* mice and mLNtx and pLNtx B6-*Il10^-/-^* mice (filled circles: colitis, filled squares: mLNtx, filled diamonds: pLNtx). Relative differences in gene expression were calculated by the comparative 2^-δCt^ method. Data were shown as mean ± 95% confidential intervals (n=3–5). **(D)** Flow cytometry analysis of single cell suspensions from mLN isolated from inflamed B6-*Il10^-/-^* mice and mLNtx and pLNtx B6-*Il10^-/-^* mice (filled circles: colitis, filled squares: mLNtx, filled diamonds: pLNtx). Plots summarize frequencies of immune cell populations (CD4^+^ T cells, CD8^+^ T cells, and CD19^+^ B cells) and stromal cell populations (Gp38^+^, CD21/35^+^, and Lyve-1^+^ stromal cells) isolated from mLN. Data were shown as mean ± 95% confidential intervals (n=3–5). Statistically significant differences are indicated by *, P< 0.05; **, P< 0.01; ***, P< 0.001; ****, P< 0.0001.

We and others already described earlier that stromal cells from pLN express a different cytokine and chemokine pattern than mLN stromal cells (18, 24). Furthermore, pLN transplanted (pLNtx) mice showed decreased antigen specific IgA^+^ B cells after cholera toxin administration compared to mesenteric lymph node transplanted (mLNtx) mice, as well as alterations in oral tolerance induction. Therefore, we transplanted pLN of B6-*Il10* ^-/-^ mice in the mesentery of B6-*Il10* ^-/-^ mice. After a regeneration phase of eight weeks, colitis in B6-*Il10^-/-^* mice was triggered by administration of 3.3% DSS in the drinking water for four days. After DSS treatment mice received water *ad libitum* for subsequent four weeks. The colon histopathological score was lower in pLNtx than in mLNtx mice (**Figure 4A**). The intestinal inflammation in mLNtx mice was characterized by moderate to marked infiltrations of immune cells up to L. muscularis, erosions of the epithelium, and moderate to marked architecture loss. Furthermore, increased IL4 levels in serum (**Figure 4B**) and increased *Tnfa* and *Il4* expression (**Figure 4C**) were detected in pLNtx mice. Although we did not find any differences in the lymphocytes compartment, we observed an increased percentage of podoplanin^+^ cells in pLNtx mice. Podoplain^+^ cells from pLNtx mice expressed less MHC class II molecules than podoplain^+^ cells from mLNtx mice (**Figure 4D**). Furthermore, we observed increased expression of activation markers, CD106 and CD54, on CD21/35^+^ cells in pLNtx mice (**Figure 4D**). Thus, pLN stromal cells seem to induce an altered immune reaction during colitis development in B6-*Il10* ^-/-^ mice, resulting in a decreased disease severity.

## Discussion

In the past 10 years, lymph node stromal cells came into focus for immunologists, as these cells were shown to influence immune response and tolerance induction. In this study we show that stromal cells could impact the development of chronic colitis via T cell induction.

The *Il10* ^-/-^ mouse model was engineered in 1993 by Kühn and colleagues (1). These mice are also widely used as a model of experimental IBD. Under conventional barrier conditions, these animals develop a chronic enterocolitis associated with anemia, splenomegaly, and weight loss. Histologically, the disease is characterized by transmural, mononuclear, and granulomatous infiltrations, crypt abscesses, erosions, or focal ulcerations. With the progression of the intestinal inflammation, thickened colon wall, hyperplasia, or loss of the crypt architecture occurs. All parts of the lower gastrointestinal tract including colon, caecum, and small intestine can be affected (25). In this study B6-*Il10^-/-^* mice showed a severe intestinal inflammation affecting the whole intestinal tract including small intestinal and colon draining mLN. Increased levels of pro-inflammatory cytokines, reduced effector T and B cells in the mLN, but also increased IgA^+^ B cells and CD4^+^ and CD8^+^ T cells in the intestine were detected. The anti-inflammatory cytokine IL10 is produced by a variety of cells including T and B cells, DC and macrophages (4, 25). IL10 is important to negatively regulate the innate as well as the adaptive immune system (26, 27). The deficiency of IL10 leads to an increased DC activity and an enhanced T effector cell induction. The excessive adaptive immune response in the *Il10* ^-/-^ model was initially identified as a Th1 response (1, 28). However, several other studies also support the contribution of a Th17 lineage (26, 29).

Besides building the backbone of the LN, SCs were shown to be involved in tolerance induction as well as inflammatory responses (7, 12, 15, 16, 18, 19, 30). LNSCs enhance the expression of genes encoding inflammatory cytokines, TLR4 signaling, and MHC class II genes after LPS stimulation (15), viral infection (31), or *Yersinia pseudotuberculosis* infection (16). In our study, we showed that mLN-derived stromal cells can play a role in the development of chronic colitis. Podoplanin^+^ cells as well as FDCs showed increased surface expression of MHC class II molecules. Moreover, strong upregulation of CD106 on podoplanin^+^ cells suggests an increased T cell-FRC contact during inflammation.

FRCs were shown to directly interact with T cells producing IL7 or CCL19/21 to promote survival (32, 33), migration, and Treg induction (19). Therefore, we hypothesized that cytokines or chemokines produced by FRCs directly activate T cells during inflammation. Chemokines such as *Ccl2*, *Ccl5*, *Ccl7*, and *Cxcl16* were shown to be expressed by lymph node stromal cells (15, 16). As shown previously, mLN consists of various lymph nodes, which drain different regions of the intestine (21, 22). Therefore, we isolated stromal cells from the smLN as well as cmLN and measured the expression of these chemokines. All chemokines were increased in stromal cells isolated from inflamed cmLN except *Ccl5*. To determine the impact of these chemokines on T cell, we isolated T cells, treated them with cytokines/chemokines, and analyzed the proliferation and cytokine secretion after 48 hours. Although we expected an activation of T cells after CD3 stimulation alone, we were not able to detect an increase in CD25 expression in all analyzed stimulation settings. We hypothesize that this could be due to the health status of *Il10* ^-/-^ mice. The isolated T cells could be activated *in vivo* due to low-level inflammation already existing in *Il10^-/-^* mice, thus, additional activation with CD3 could not be achieved in the performed stimulation assay. Stimulation with CXCL16 and CCL7 activated T cells as observed in increased proliferation or CD25 expression, respectively. CCL7 was shown to induce migration of various leukocytes including monocytes and lymphocytes (34), while soluble CXCL16 is a strong chemoattractant for CXCR6^+^ T cells (35). Furthermore, CCL7 and CXCL16 were found to be increased in the intestine of IBD patients as well as in mouse models of experimental colitis (18, 36). Both chemokines seem to be involved in T cell activation and migration leading to exacerbated intestinal inflammation. However, stimulation with most of the cytokines/chemokines did not activate T cells, suggesting that there is another cell population interacting with LNSCs. Potential candidate lineage could be DCs, as stromal cells were shown to induce tolerogenic DCs in mLN (16). Stromal cells from different draining areas can influence immune cells in a different way. Using a lymph node transplantation model, in which pLN were transplanted into mesentery, we previously showed that oral tolerance in pLNtx mice is induced via B cells in contrast to Treg induction in endogenous mLN (12). Moreover, mLN transplanted into the periphery are more efficient in Treg induction as the endogenous LN (19). Therefore, this model enables us to analyze the influence of stromal cells on immune response. In this study, pLN transplanted into the mesentery resulted in increased gene and protein expression of IL4, and a decreased disease severity. IL4 is a Th2 cytokine that inhibits T cell differentiation into Th1 and Th17 phenotype. As colitis in *Il10^-/-^* mice is dependent on Th1 and Th17 immune responses, increased expression of IL4 can act as a negative regulator of inflammation. The protective effect of IL4 has been shown in murine models of rheumatoid arthritis and diabetes (37–39). However, *Il4^-/-^Il10^-/-^* double deficient mice showed decreased colitis induction after DSS treatment (40) indicating that IL4 alone is not sufficient to reduce chronic colitis severity. Further studies need to be performed to evaluate the role of IL4 in chronic inflammation. Nevertheless, pLN stromal cells induce an altered immune response during colitis development in B6-*Il10^-/-^* mice indicating importance of imprinting processes. As mLN stromal cells acquire their specific immune response induction pattern early during neonatal microbiota colonization, lymph nodes from skin draining area develop different expression patterns (16).

In conclusion, lymph node stromal cells are activated during colitis development. Cytokines expressed by colitogenic stromal cells can influence T cell activation *in vitro*. However, stromal cells from lymph nodes of different draining areas regulate induction of immune responses toward neonatally imprinted processes.

## Data Availability Statement

The original contributions presented in the study are included in the article/**Supplementary Material**. Further inquiries can be directed to the corresponding author.

## Ethics Statement

The animal study was reviewed and approved by Lower Saxony State Office for Consumer Protection and Food Safety.

## Author Contributions

MBu and UB conceived experiments. MBu, PPP, and MBo performed experiments. AB, MBu, SB, and MBo analyzed results. MBu and MBa interpreted results and wrote the manuscript. All authors contributed to the article and approved the submitted version.

## Funding

The work was supported by the German Research Foundation (SFB621/A10) to UB and by the Hannover Medical School project funding program, Hochschulinterne Leistungsförderung HiLF to MBu.

## Conflict of Interest

The authors declare that the research was conducted in the absence of any commercial or financial relationships that could be construed as a potential conflict of interest.

## Supplementary Material

Supplementary Figure 1 Surface staining of total cell populations of the mLN and small intestine from B6-*Il10^-/-^* mice was performed and analyzed by flow cytometry. Gating strategy for mLN cells shows CD3+ cells, CD19+ cells gated from the leukocyte gate. CD4+ and CD8+ cells were gated from CD3+ cells. Homing marker CCR9 and LPAM1 were analyzed from CD19+ or CD4+ and CD8+ cells. Gating strategy for cells isolated from small intestine shows CD3+ cells, CD19+ cells, IgA+ cells, MHCII/CD11c+ cells (DC), and MHCII/F4/80+ cells (macrophages) gated from the leukocyte gate. CD4+ and CD8+ cells were gated from CD3+ cells.

Supplementary Figure 2 Gating strategy for stromal cells analysis. From living cells, CD45- cells were gated and subpopulations were identified using podoplanin+ (gp38), CD21/CD35+ (FDC), and Lyve-1+ (lymphatic endothelial cells) cells. Surface marker MHCII, CD106, and CD54 were gated from gp38+ cells or CD21/CD35+ cells.

Supplementary Figure 3 Gating strategy for cytokine stimulated T cells. T cell subpopulations were identified using CD3, CD4, and CD8 antibodies. CFSE as well as CD25+ cells and CCR9+ cells were gated from CD3/CD4+ cells and CD3/CD8+ cells.
