# Supplementary figures and images for: Lymph Node Stromal Cells From Different Draining Areas Distinctly Regulate the Development of Chronic Intestinal Inflammation
Source: Front Immunol. 2021 Feb 16;11:549473. doi: 10.3389/fimmu.2020.549473 (PMC7921801; doi:10.3389/fimmu.2020.549473)

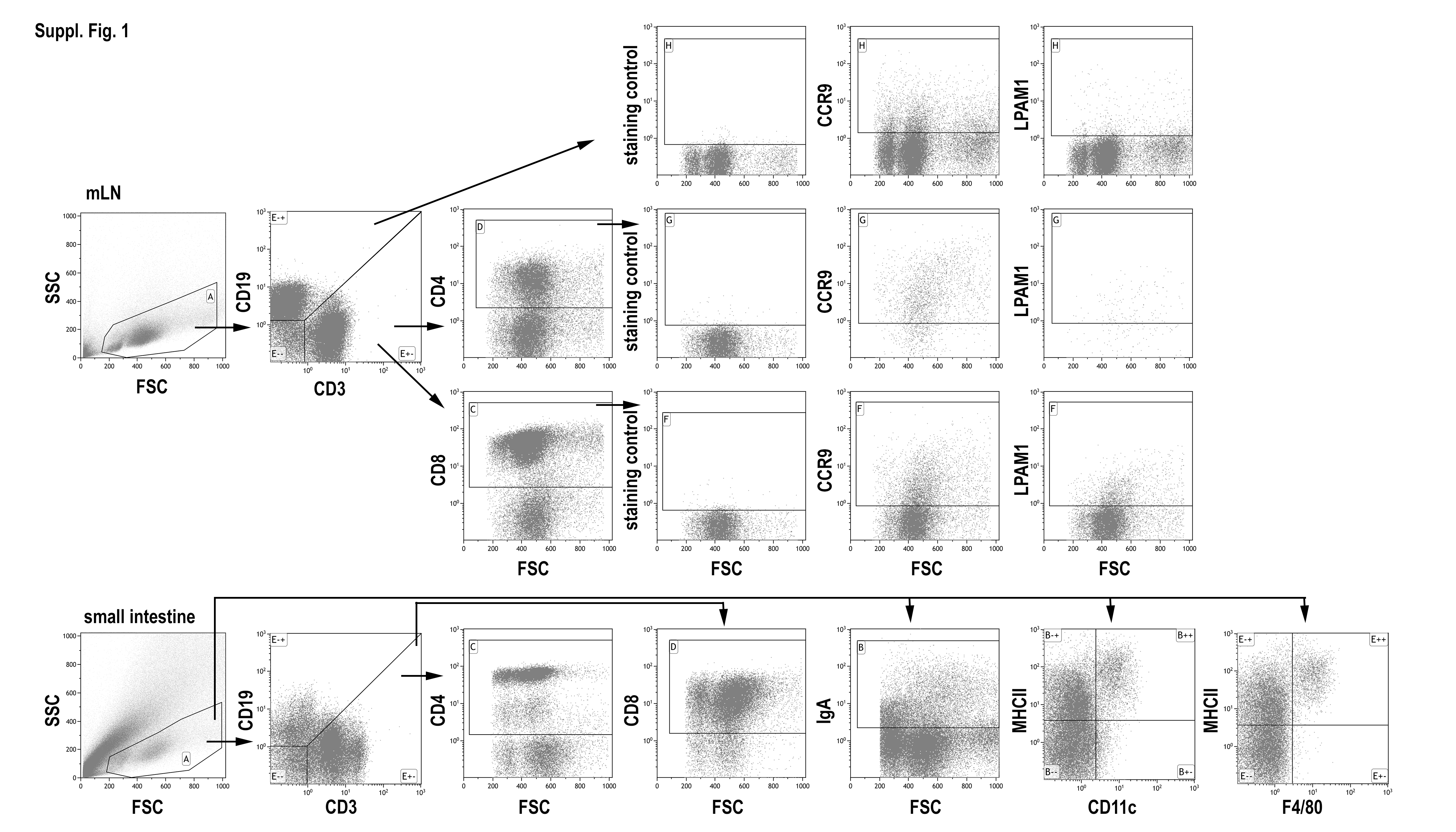

Supplement: Supplementary Figure 1 — Surface staining of total cell populations of the mLN and small intestine from B6-Il10-/- mice was performed and analyzed by flow cytometry. Gating strategy for mLN cells shows CD3+ cells, CD19+ cells gated from the leukocyte gate. CD4+ and CD8+ cells were gated from CD3+ cells. Homing marker CCR9 and LPAM1 were analyzed from CD19+ or CD4+ and CD8+ cells. Gating strategy for cells isolated from small intestine shows CD3+ cells, CD19+ cells, IgA+ cells, MHCII/CD11c+ cells (DC), and MHCII/F4/80+ cells (macrophages) gated from the leukocyte gate. CD4+ and CD8+ cells were gated from CD3+ cells. [file Image_1.tif]

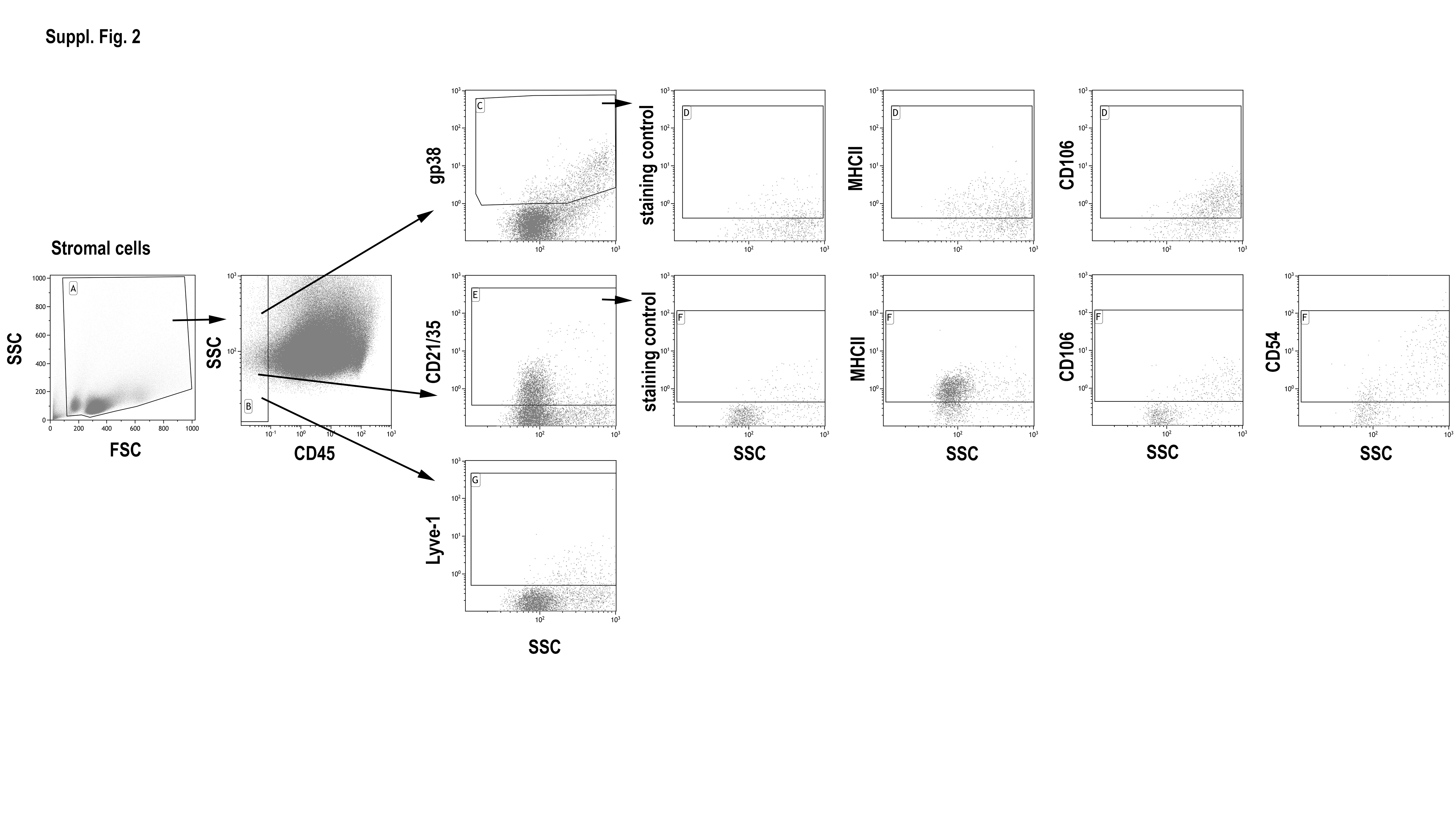

Supplement: Supplementary Figure 2 — Gating strategy for stromal cells analysis. From living cells, CD45- cells were gated and subpopulations were identified using podoplanin+ (gp38), CD21/CD35+ (FDC), and Lyve-1+ (lymphatic endothelial cells) cells. Surface marker MHCII, CD106, and CD54 were gated from gp38+ cells or CD21/CD35+ cells. [file Image_2.tif]

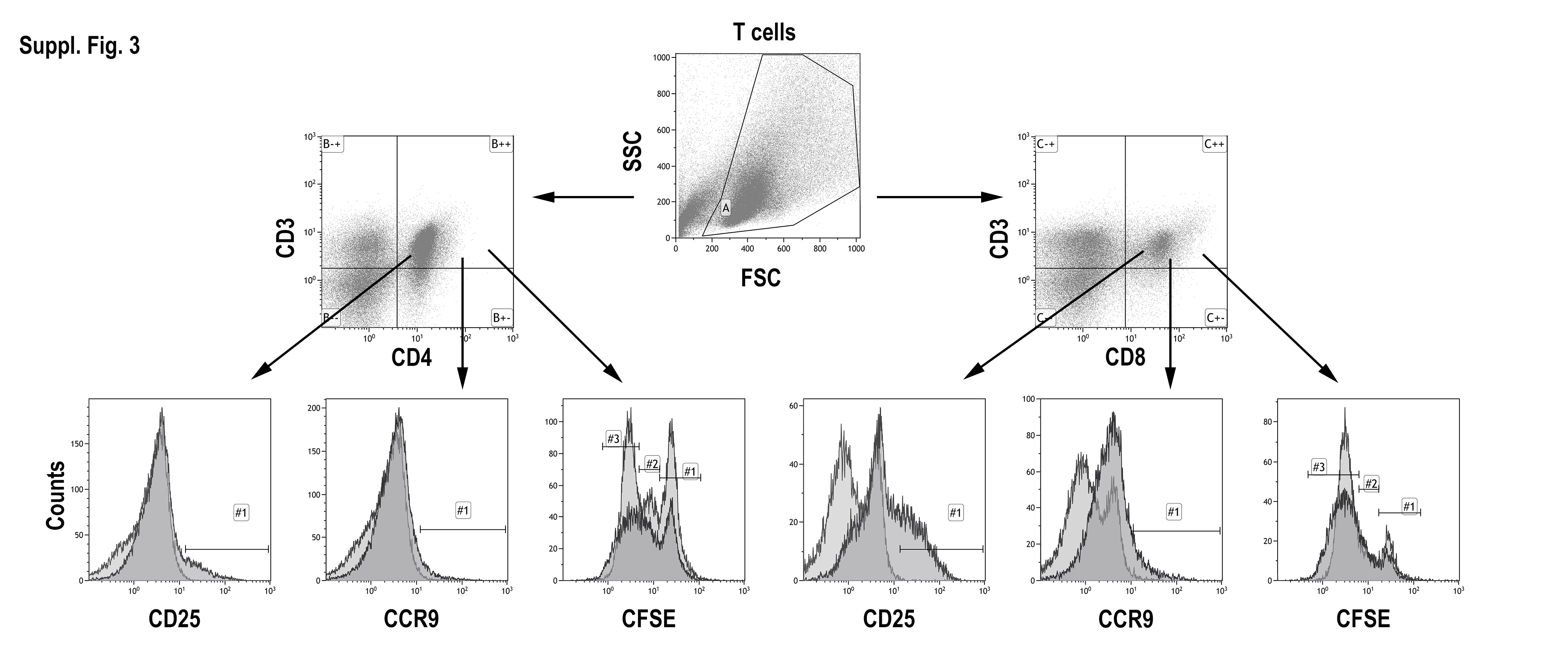

Supplement: Supplementary Figure 3 — Gating strategy for cytokine stimulated T cells. T cell subpopulations were identified using CD3, CD4, and CD8 antibodies. CFSE as well as CD25+ cells and CCR9+ cells were gated from CD3/CD4+ cells and CD3/CD8+ cells. [file Image_3.tif]
